# Investigation of Surface Layers on Biological and Synthetic Hydroxyapatites Based on Bone Mineralization Process

**DOI:** 10.3390/biomimetics8020184

**Published:** 2023-04-28

**Authors:** Kazuto Sugimoto, Yanni Zhou, Tania Guadalupe Peñaflor Galindo, Reo Kimura, Motohiro Tagaya

**Affiliations:** 1 Department of Materials Science and Technology, Nagaoka University of Technology, Kamitomioka 1603-1, Nagaoka, Niigata 940-2188, Japan; 2 Department of Materials Science and Bioengineering, Nagaoka University of Technology, Kamitomioka 1603-1, Nagaoka, Niigata 940-2188, Japan; 3 General Department, National Institute of Technology, Nagaoka College, 888 Nishikatagai, Nagaoka, Niigata 940-8532, Japan

**Keywords:** hydroxyapatite, non-apatitic layer, hydration layer, surface properties, biocompatibility, bone tissue formation, mineralization

## Abstract

In this review, the current status of the influence of added ions (i.e., SiO_4_^4−^, CO_3_^2−^, etc.) and surface states (i.e., hydrated and non-apatite layers) on the biocompatibility nature of hydroxyapatite (HA, Ca_10_(PO_4_)_6_(OH)_2_) is discussed. It is well known that HA is a type of calcium phosphate with high biocompatibility that is present in biological hard tissues such as bones and enamel. This biomedical material has been extensively studied due to its osteogenic properties. The chemical composition and crystalline structure of HA change depending on the synthetic method and the addition of other ions, thereby affecting the surface properties related to biocompatibility. This review illustrates the structural and surface properties of HA substituted with ions such as silicate, carbonate, and other elemental ions. The importance of the surface characteristics of HA and its components, the hydration layers, and the non-apatite layers for the effective control of biomedical function, as well as their relationship at the interface to improve biocompatibility, has been highlighted. Since the interfacial properties will affect protein adsorption and cell adhesion, the analysis of their properties may provide ideas for effective bone formation and regeneration mechanisms.

## 1. Introduction

Bone grafting is one of the surgical treatments for bone defects caused by osteoporosis, bone tumors, and bone atrophy in dental implants, and is generally performed by using autologous or allogeneic bone grafts, in which the bone is taken from the patient’s own or a compatible donor, and then transplanted into the defect site. However, it presents some problems, such as increased burden on patients due to surgery and limitation of the amount of bone used [1,2,3,4]. Synthetic bone has recently been produced as an alternative to autogenous bone to address these problems. Calcium phosphate (CP), hydroxyapatite (HA), ceramics, CP cements, and bioactive glass are generally known as synthetic bone materials [5,6,7]. Synthetic bone has the advantages of high biocompatibility, fewer and minimal requirements for surgery, and low risk of inflammation; however, it is unable to induce cells associated with bone metabolism from the surrounding tissue after replacing a bone defect. Accordingly, the improvement of the ability to form new bone has been researched. Therefore, it is important to elucidate the mechanism of biological bone formation and to create synthetic bone that resembles biological bone.

**Figure 1:**
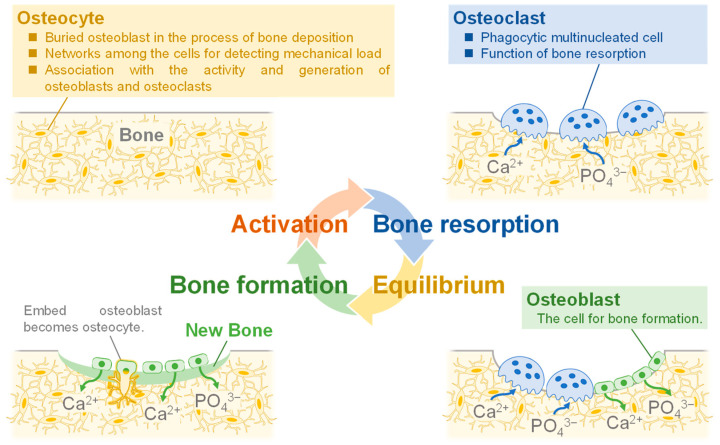
Illustration of the process of bone formation in vivo.

Biological bone is composed of an organic matrix, reinforced by the deposition of inorganic salts consisting mainly of Ca^2+^ and PO_4_^3−^, with 30 wt.% of organic matrix and 70 wt.% of inorganic salts. Biological bone is metabolically active, with “bone resorption”, the destruction of old bone, and “bone formation”, the creation of new bone. This cycle of bone metabolism is physiologically important, and its functions are to replace brittle old bone and to strengthen areas subjected to load [8,9]. Figure 1 shows the bone formation process. Osteocytes are derived from osteoblasts and are formed by the incorporation of osteoblasts into the bone matrix [10]. Osteocytes act as mechanosensory cells because they build networks by joining small tubules together [11]. Due to their function, they are thought to be the cells that detect the mechanical loading of bone and are involved in the activity and generation of osteoblasts and osteoclasts [12]. Bone resorption is carried out by phagocytic multinucleated cells called osteoclasts. These cells originate from monocytes or monocyte-like cells produced in the bone marrow [13]. Bone resorption occurs when osteoclasts come into contact with bone. During this process, proteolytic enzymes released from osteoclast lysosomes dissolve the organic substrate of bone. This mechanism releases several acids, including citric and lactic acids, from mitochondria and secretory vesicles. This phenomenon results in the dissolution of bone composed of Ca^2+^ and PO_4_^3−^ [14]. On the other hand, osteoblasts are responsible for bone formation. The mechanism of bone formation begins with the secretion of collagen molecules and substrate substances by osteoblasts. Subsequently, osteoblasts form collagen fibers from the collagen molecules to form allogeneic bone, which incorporates some of the osteoblasts to become osteocytes [15]. Within a few days of the formation of the analogous bone, amorphous calcium phosphate (ACP) begins to precipitate on the surface of the collagen fibers. The precipitated ACP is converted to HA over a period of weeks to months, during which atoms are added or replaced [16,17]. A few percent of HA remains amorphous and is more rapidly absorbed when Ca^2+^ is needed in the extracellular fluid. This is the process of bone formation. Also, in focusing on cells, they have cell-adhesive proteins called integrins on their surfaces. In these cells, the adhesion function of integrins would be carried out by peptide ligands [18]. Arg-Gly-Asp (RGD) is a type of peptide ligand that has been shown to promote osteoblast adhesion [19,20]. In the case of the RGD modified with hyaluronan, it has been reported to stimulate cell adhesion [21]. Glycine-histidine-lysine (GHK) is also the peptide ligand found in osteonectin that has been reported to enhance osteogenic differentiation [22]. Bioceramics that could facilitate this process have not yet been synthesized, which is a challenge in the development of synthetic bone.

As mentioned above, for preparing specific materials that promote bone formation and are compatible with both bone replacement and therapy, the following conditions are required:

(1) Bioceramics that resemble biological bone [23].
(2) Bioceramics with low crystallinity [24].
(3) Bioceramics of nanocrystalline structure [25,26,27].

Bioceramics that fulfil these conditions can be used to replace bone defects without toxicity to the bone, and their high bioactivity can elute bone-like components at the interface between the bone and the bioceramics to promote rapid adhesion between the bone and the bioceramics, thus facilitating treatment and allowing early treatment of bone defects [28].

In this review, the current status and issues of bone defect sites in the human body are explained based on examples of conventional HA, and the necessity of regenerative functions in addition to bone defect sites for biomedical applications is proposed. The characteristics and properties of HA substituted with heterogeneous ions such as silicate, carbonate, and other elemental ions are also explained. The importance of the surface characteristics of HA and its components, the hydration layers, and the non-apatitic layers for the effective control of biomedical function, as well as their relationship at the interface to improve biocompatibility, are highlighted. The possibility of a surface layer being formed between HA and its components is also proposed.

## 2. Hydroxyapatite (HA) Substituted with Other Ions

### 2.1. Characteristics of HA

CP is composed of Ca^2+^ and PO_4_^3−^ or P_2_O_7_^4−^ and is classified as a bioceramic. Table 1 shows the classification of CP compounds. It is classified according to the Ca/P molar ratio, which is the ratio of Ca^2+^ to PO_4_^3−^. Six types are present in hard tissues in vivo, with Ca/P molar ratios in the range of 1.0–1.67 [29]. The bioactivity of it is related to their crystalline structure, porosity, and dissolution rate, which are controlled by changing the parameters for various biomedical applications. In particular, the porosity of HA containing nanoparticles or nanopores is expected to function as a drug delivery system [30,31]. The controlled parameters are known to be dominantly influenced by the Ca/P molar ratio. Among the CPs, HA is the major component of bone and has been widely studied by controlling the parameters.

**Figure 2:**
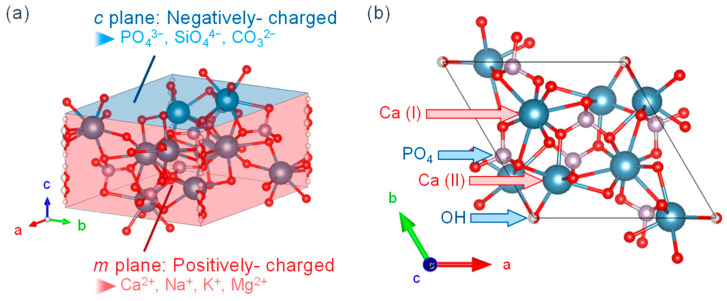
HA crystalline structure from the views of (**a**) overall and (**b**) [001].

Figure 2 shows the crystalline structure of HA, with (a) the overall view and (b) the view from [001]. HA has a Ca/P molar ratio of 1.67 and a chemical composition of Ca_10_(PO_4_)_6_(OH)_2_. The crystalline structure is hexagonal, the space group is P6_3_/m, and the unit cell size is a = 0.94 nm and c = 0.69 nm. The calcium in the structure is classified into Ca(I) (columnar Ca) and Ca(II) (axis Ca), where the Ca(I) is aligned parallel to the *c*-axis and the Ca(II) surrounds the *c*-axis at the four corners of each unit cell where hydroxyl groups are present [32]. It is also known that HA has a high ion exchange capacity. The Ca^2+^ is substituted by Na^+^, K^+^, Mg^2+^, etc.; the PO_4_^3−^ by SiO_4_^4−^, CO_3_^2−^, etc.; and the OH^−^ by F^−^, Cl^−^, etc. [33]. These ions are thought to exist within the crystal and on the surface. They exist by substitution with Ca^2+^, PO_4_^3−^, and/or OH^−^ when they are present inside the crystal and by reaction with functional groups such as P-OH and Ca-OH exposed on the surface when they are present on the surface.

Amorphous HA can be synthesized by some synthetic methods; highly crystalline and stable HA can be obtained by calcination of amorphous HA. HA is known to have high biocompatibility since it is contained in biological hard tissues. Therefore, it is used in biomaterials such as artificial bones and implants, and as a fixation layer for chromatography due to its high protein adsorption capacity [34,35,36]. Recently, its biocompatibility and protein adsorption capacity have been investigated for use as a drug delivery system [37,38]. 

The composition, structure, and morphology of HA vary greatly depending on the synthetic method. The techniques for synthesizing HA include the hydrothermal method, chemical precipitation method, emulsion method, and solid phase reaction method [39,40,41,42,43]. In human bodies, HA is synthesized by the biomineralization process. Recent studies on biomineralization described the biological formation of HA and its nucleation in body fluids to finally be self-assembled into complex structures such as teeth and bones [44]. The HA obtained by the biomineralization process promotes osteoblast adhesion, proliferation, and osseointegration [45,46]. The method for precipitating HA by simulating the biological fluid environment has been studied. Among these synthetic methods of producing HA, except for the hydrothermal method, calcium-deficient HA (CDHA) is easily obtained. Furthermore, ACP can be easily precipitated in aqueous solutions of Ca^2+^ and PO_4_^3−^ at pH values above 9 for synthetic HA. The obtained ACP can be converted to HA by the recrystallization method [33,47,48]. 

### 2.2. Synthesis of HA to Enhance Its Biological Functions: Ion Substitution with Different Elements

HA in biological hard tissues is substituted with various elemental ions, such as carbonate and silicate ions, that work on the concentration, size, and type of action on the HA crystal lattice to enhance its physicochemical properties by altering its electron density and surface conditions [49]. Table 2 shows the different elemental ions that can be substituted in HA. These substitutions can be a cationic ion (with Ca^2+^) or an anionic ion (with PO_4_^3−^ and OH^−^). Ionic substitution affects lattice parameters, crystallinity, surface charge, and morphology. The Ca^2+^ sites are mainly replaced by alkali metals and alkaline earth metals, and partially by transition metals. The lattice constants of the HA crystal structure are changed by these substitution ions, which is mainly attributed to the deficiency of OH^−^ and Ca^2+^ and the change of ionic radius. For example, Mg^2+^, Sr^2+^, Mn^2+^, and Zn^2+^ ions increase the a-axis and the c-axis, while Na^+^, SiO_4_^4−^, CO_3_^2−^ (type B), and F^−^ ions decrease the a-axis and increase the c-axis, CO_3_^2−^ (type A) and Cl^−^ increase the a-axis and decrease the c-axis, and K^+^ decreases the a and c-axis [49].

The functions performed by the other elemental ions that are substituting in HA in biological bone affect various components of the bone metabolic process. The important phenomena in bone formation and implantation, namely osteoinduction, osteoconduction, and osseointegration, have been investigated in the previous study [50,51]. Osteoinduction is the process by which primitive, undifferentiated pluripotent cells are stimulated in some way to grow into the osteogenic cell lineage and induce osteogenesis [51,52]. In addition to osteoblasts and osteoclasts, the bones and surrounding tissues contain many undifferentiated cells. These undifferentiated cells have been reported to develop into osteocytes over time and are crucially important for bone healing and implant fixation [53]. The other elemental ions have been reported to promote bone formation and other processes by regulating the expression of genes and proteins involved in various stages of osteogenic differentiation [54,55,56,57]. This is a fundamental biological mechanism that occurs regularly in bone defect treatment and implantation. Osteoconduction refers to the ability of biomaterial surfaces to grow bone and is the process of inducing the adaptation of different biomaterial surfaces to the biological body. Since bone growth at the implant surface is dependent on the action of differentiated osteocytes, it can be considered that osteoconduction is dependent on osteoinduction. Moreover, various types of bone growth factors are required for bone formation and bone growth, including osteoconduction, which cannot occur without an adequate blood supply [58]. Osteoconduction in implants also depends on the biomaterial used and its response. In the case of metallic materials, there are reports showing that osteoconduction is not possible with Ag and Cu while it is possible with Ti and other materials [50]. Osseointegration is defined as the direct contact between bone and implant at the order-level by an optical microscope, as well as between the implant and bone via the cells or other biological tissues, with the result that the bone tissue can be formed at the bone-implant interface and the implant is directly fixed [59,60]. Osseointegration is not an isolated phenomenon; it depends on bone induction and osteogenesis. Therefore, materials that cannot promote bone growth cannot undergo osseointegration. These phenomena are interrelated, and the development of biomaterials that contribute to these phenomena is considered very important in the field of implants.

The substitution of other elemental ions in HA can enhance osteoblast differentiation, osteoinductive, osteoconductive, and osseointegration functions, such as the release of substituted ions due to reduced solubility of HA. Substitution of Cu^2+^, Mg^2+^, and SiO4^4−^ enhances osteoinduction, whereas substitution of Na^+^, CO_3_^2−^, and Cl^−^ enhances osteoinduction [61,62,63]. Furthermore, HA substituted with Zn^2+^ and Sr^2+^ has various biological functions, such as enhancement of bone formation and suppression of bone resorption and osteoporosis. It has been reported that SiO_4_^4−^-substituted HA improves osseointegration properties and dissolution rate. F^−^-substituted HA was used to treat osteoporosis; however, excessive amounts of F^−^ may cause osteosclerosis and other diseases [64,65,66,67,68,69,70]. The combination of these different elemental ion substitutions is considered important for the synthesis of HA, which retains its beneficial properties for bone formation. 

### 2.3. Structure and Properties of HA with Silicate Ion Substitution

**Figure 3:**
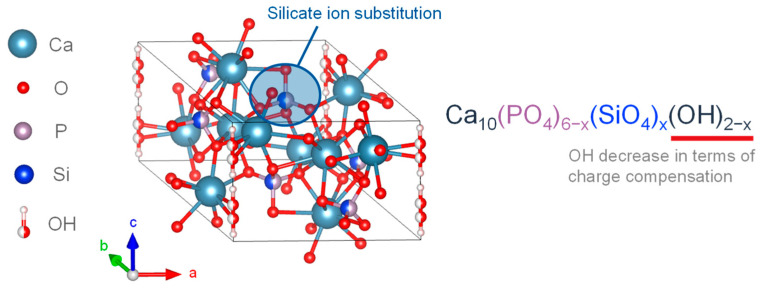
Silicon-substituted HA crystalline structure.

Studies have found that the human body contains about 1–2 g of silicon (Si), and biological bone contains 36 ppm [71,72]. In mice and rats, 0.5 wt.% Si is present in the active growth points of bones [73], and a lack of Si in the diets of mice and rats leads to abnormal bone growth and cranial deformation [74]. The presence of Si affects bone growth. In vitro studies demonstrated that the ingestion of Si, in the form of orthosilicate, enhanced collagen I synthesis and promoted osteoblast differentiation [75]. On the other hand, in synthetic bioactive ceramics, such as bio-glasses and apatite-wollastonite containing SiO_2_, the reactivity of the crystalline surface containing Si affects its bioactivity [76]. SiO_4_^4−^-substituted HA particles have also been produced in various forms and used as coatings for titanium implants, showing improved osseointegration properties [66,68,77]. A synthetic porous SiO_4_^4−^-substituted HA with the trade name Actifuse TM has been successfully used as a bone replacement material in patients with level 1–2 lumbar degenerative disease and was found to be as effective as an autologous bone graft [78]. Figure 3 shows the crystal structures of previously described SiO_4_^4−^-substituted HA [76]. Silicic acid was present in HA in the form of an anionic substitution. Gibson et al. successfully synthesized the single-phase SiO_4_^4−^-substituted HA with SiO_4_^4−^ substitution (0.4 wt.%) into the PO_4_^3−^ site of HA with a precipitation reaction using calcium hydroxide as the calcium source, orthophosphoric acid as the phosphoric acid source, and silicon tetraacetate as the SiO_4_^4−^ source. The compositional formula was proposed as shown in Equation (1) [76].

Ca_10_(PO_4_)_6−x_(SiO_4_)_x_(OH)_2−x_ (1)

The experimental results of SiO_4_^4−^ substitution of HA showed that the OH^−^ group decreased with the SiO_4_^4−^ substitution, and the SiO_4_^4−^substitution caused changes in the crystal structure and chemical composition, including a decrease in the *a*-axis and an increase in the *c*-axis [76]. Bianco et al. synthesized SiO_4_^4−^-substituted α-TCP in a mixed phase with 1.26 wt.% SiO_4_^4−^ and CO_3_^2−^ with a precipitation reaction using calcium hydroxide as the calcium source, orthophosphoric acid as the phosphoric acid source, and tetraethoxysilane (TEOS) as the SiO_4_^4−^ source. As a result, these particles were found to have an increased *a*-axis and *c*-axis [79]. In an example of synthesizing SiO_4_^4−^-substituted HA by varying the added amount of SiO_4_^4−^ from 0.8–5.0 wt.%, it was confirmed that the incorporated concentration of SiO_4_^4−^ was up to 1.5 wt.%, and the SiO_4_^4−^-substituted HA exceeding 1.5 wt.% contained ACP with amorphous silica [80].

### 2.4. Structures and Properties of Carbonate Ion Substituted HA

Carbonate ion (CO_3_^2−^) is present in biological hard tissues, such as biological bone and dentin tissue, in an amount of 2.3−8.0 wt.%, in the form of substitution with PO_4_^3−^ and OH^−^ in HA [81,82]. The crystalline structure of CO_3_^2−^-substituted HA is similar to that of HA in biological hard tissues. It is suggested to play an important role in bone metabolism and is expected to be used as a bone replacement material [81,82,83,84,85].

**Figure 4:**
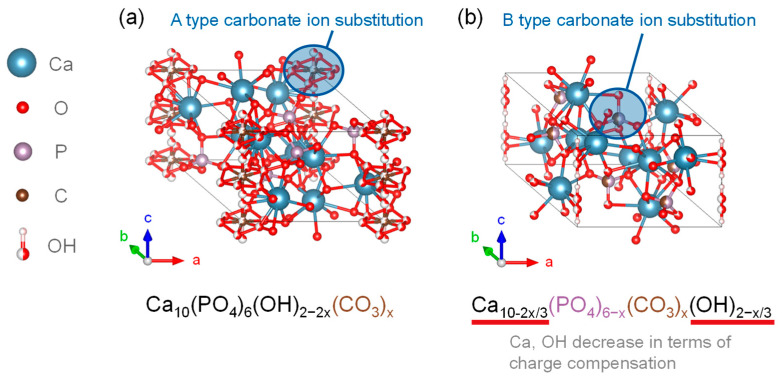
Carbonate-substituted hydroxyapatite crystalline structures of (**a**) CAP and (**b**) CHA.

Figure 4 shows the crystalline structure of two types of CO_3_^2−^-substituted HA. It is an anionic substitution and has two different forms depending on the substitution site [86,87,88]. The A-type carbonate HA is in Figure 4a. It is called carbonate apatite (CAP), and is the substitution form in which CO_3_^2−^ is substituted with the OH site of HA. The chemical composition is expressed by the formula in Equation (2).

Ca_10_(PO_4_)_6−x_(OH)_2−2x_(CO_3_)_x_ (2)

In general, CAP can be synthesized by heating HA to approximately 1000 °C in a carbon dioxide atmosphere [89]. The lattice parameters of CAP prepared by Walleyes showed an increase in the *a*-axis and a decrease in the *c*-axis in X-ray diffraction. It was reported that for every 1 wt.% substitution of CO_3_^2−^ in CAP, the *a*-axis of the lattice parameter was lengthened by 0.0025 nm, with an upper limit of up to 4.4 wt.% [90,91].

The B-type carbonate-substituted HA (CHA) shown in Figure 4b is a substitutional form in which CO_3_^2−^ was substituted at the PO_4_^3−^ site of HA, and the chemical compositions of the substitutional formulas are given in Equations (3) and (4) [92].

Ca_10−2x/3_(PO_4_)_6−x_(CO_3_)_x_(OH)_2−x/3_ (3)

Ca_10−x/2_(PO_4_)_6−x_(CO_3_)_x_(OH)_2_ (4)

CHA remains charge neutral due to the loss of Ca^2+^ and OH^−^ upon CO_3_^2−^ substitution. For each 1 wt.% substitution of CO_3_^2−^, the *a*-axis of the lattice parameter was shortened by 0.00006 nm and the *c*-axis was elongated, which could be up to the maximum inclusion content of 22.2 wt.% [91]. The AB-type carbonate-ion-substituted HA, in which carbonate ions were substituted at both the PO_4_^3−^ and OH^−^ sites of the HA crystalline structure, was also present [93]. The HA showed enhanced dissolution properties in vitro and in vivo, and had higher osteoconductivity than the stoichiometric HA. The increased CO_3_^2−^ content in HA results in the formation of CaO and β-TCP phases by calcination at temperatures where CO_3_^2−^ decomposes, and the mechanical tests showed that CAP had similar strength to that of stoichiometric HA [93].

In dentistry, it was observed that an increase in CO_3_^2−^ content increases the dissolution characteristics of HA in weak acids. This property has been reported to preferentially dissolve tooth enamel, the surface material of teeth, in the initial stages of dental caries, leading to the progression of caries [94,95]. Depending on the progression of caries, secondary caries and tooth erosion may occur; therefore, proper control of CO_3_^2−^ ion substitution is important [96,97]. Thus, the changes caused by HA dissolution in bone due to CO_3_^2−^ ions should be studied from the viewpoints related to bone remodelling.

## 3. Surface Layers on HA

### 3.1. Hydration Layer

As the bioceramics come into the contact with water molecules, the water molecules are adsorbed onto the surfaces and the hydration layers are formed. The formation of the hydration layer is caused by various factors, including the interactions between the bioceramic surfaces, ions, and water molecules. The interactive states play an essential role in the subsequent biological reactions [98,99,100]. 

**Figure 5:**
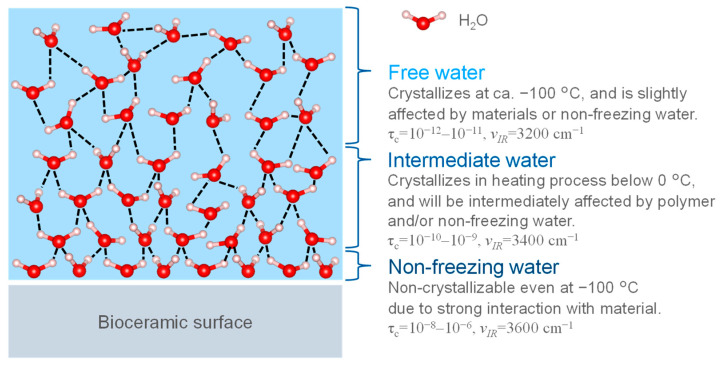
The three types of hydration layers. τ_c_: relaxation time of water molecule motion by solid state NMR measurement. ν_IR_: IR absorption wavenumber.

Figure 5 shows the three hydration layers on the bioceramic surface. In most of the studies, the hydration layer consists of three layers: unfrozen water, intermediate water, and free water, and it has been thought that the cell-adhesive proteins are affected by retaining these layers [101]. Non-freezing and intermediate water are unable to form hydrogen bonds due to strong interactions with the bioceramic surface, thus, water molecules remain in a state where they can move and are difficult to freeze. Free water does not directly interact with the bioceramic surfaces and behave similarly to bulk water, forming hydrogen bonds with the surrounding H_2_O. Below 0 °C it freezes, thus stopping the molecular motion. The difference between the three layers was evaluated in terms of the heat balance during the freezing and melting of water by differential scanning calorimetry (DSC) and the mobility of water molecules by ^1^H-NMR [102]. The difference between the three layers was due to their interaction acting on the bioceramic surfaces, which modifies the melting point and the mobility. Based on the thermal value at each transition of DSC, non-freezing water was defined as water that does not freeze at −100 °C; intermediate water was water that froze at temperatures lower than 0 °C during the temperature increase process; and free water was water that froze at temperatures below 0 °C and crystallized at −100 °C [102]. The layer structure was defined as water that was crystallized at temperatures below 0 °C and crystallizes at −100 °C [103]. The structure of the hydration layer rearranges on femto- to picosecond time scales, and liquid water is an amorphous structure with a disordered network on very short time scales; however, it has the randomness of a liquid on longer time scales. In ^1^H-NMR measurements, the relaxation times of non-freezing water, intermediate water, and free water were 10^−8^–10^−6^ s, 10^−10^–10^−9^ s, and 10^−12^–10^−11^ s, respectively, indicating stronger interactions with the bioceramics as well as lower mobility [102]. In the FT-IR spectra, the layer state could be evaluated by separating the absorption bands of the stretching vibration of the hydroxyl groups (3600 cm^−1^, 3400 cm^−1^, 3200 cm^−1^) corresponding to non-freezing water, intermediate water, and free water, respectively [102]. A variety of other analytical methods were used to evaluate the hydration structure (i.e., hydrogen molecular bonding state and mobility). However, the layer on the HA surface has not been studied in detail, thus it is necessary to evaluate the layer bonded to the surface as an integral part. 

### 3.2. Non-Apatitic Layer on HA Surface

**Figure 6:**
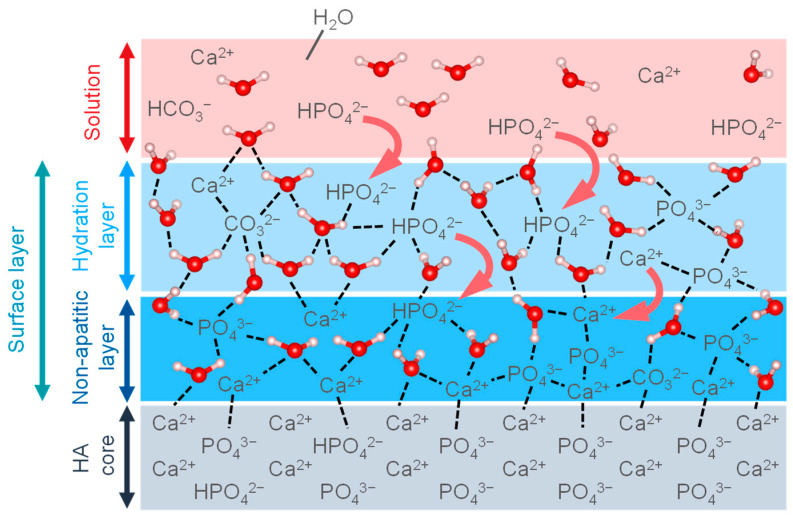
Illustration of the surface layers on HA core.

Figure 6 shows a model of the surface layer bounded with the HA. Biological apatite in naturally calcified tissue is formed in vivo in an aqueous environment at room temperature. Thus, recent studies of HA have focused on HA prepared by wet methods, which can synthesize HA similar to the in vivo environment in order to mimic biological apatite. The detailed surface structure of the HA synthesized by wet methods has not been fully elucidated; however, it is predicted that it will be a low-crystalline surface, and such a structure is being studied by spectroscopic methods such as FT-IR and NMR, which are sensitive to perturbations of the local ionic environment. FT-IR spectra show an absorption band in the 680–480 cm^−1^ region corresponding to the non-apatitic layer on the HA surface, which is assigned to PO_4_^3−^ and HPO_4_^2−^ [103]. The coordination environment of phosphate and calcium ions and the interaction of these ions were investigated by NMR spectroscopy, indicating that the HA particles are composed of a highly crystalline core and a non-apatite layer composed of ACP [104]. The layer is highly reactive due to its unstable structure composed of divalent ions such as Ca^2+^, HPO_4_^2−^, and CO_3_^2−^, etc. In particular, it has been shown from ab initio calculations that the reaction with H_2_O exhibits moderate Lewis acidity due to the strong bonding of the exposed ions in the non-apatitic layer (e.g., Ca^2+^, HPO_4_^2−^, CO_3_^2−^, etc.) due to electrostatic interactions with the H_2_O [105,106]. The ions in the layer are organized in a geometric configuration and are stabilized by a structured hydrogen bonding network of the hydration layer [106]. In addition to this reaction, the layer is believed to contribute to the growth of HA nuclei, ion exchange, and the adsorption of organic molecules. The reactivity of the layer is thought to play a significant role in biocompatibility, and further research is needed. 

### 3.3. Relationship between the Surface Layer and Biocompatibility

**Figure 7:**
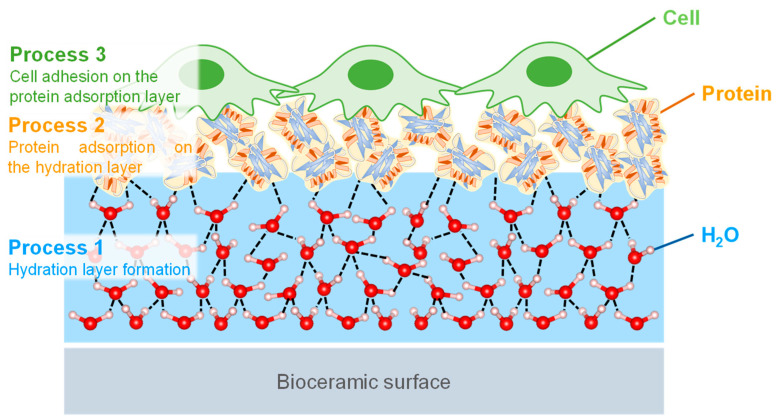
Illustration of the cell adhesion processes of (1) the hydration layer formed on the bioceramic surface, (2) protein adsorption on the hydration layer, and (3) cells that recognize and adhere to the protein adsorption layer.

The biocompatibility of HA is highly related to the cell adhesion behavior, and the cell-bioceramic interfaces are formed in vivo, as shown in Figure 7 [107,108]. Firstly, the cell adhesion occurs through three processes upon implantation in the body. In the process, H_2_O and ions in body fluid adsorb on the surfaces, forming the hydration layer. Here, the amount of H_2_O, the concentration and type of ions in the vicinity of the bioceramics are important factors for the next processes. In the second process, the proteins are adsorbed onto the hydration layer, and the protein adsorption is saturated to form the protein adsorption layer. The type and orientation structure of the adsorbed protein in this process determine the function of the attached cells. In the third process, the cells are adhered to the adsorbed protein layer and proliferate with their spreading. The layers formed by the first and second processes determine the cell behavior. Thus, it is important to control the first process and then evaluate the protein adsorption layer for the desired cell adhesion properties in order to consider biocompatibility [109,110]. The highly-reactive surface layer on HA is known to interact strongly with the substances in surrounding aqueous solutions [111,112,113]. The interactions are supported by ion mobility, ion exchange capacity, and molecular adsorption [114,115,116]. The interfacial phenomena induce the combination between HA surface layer and water, such as the dissolution and deposition of ions and the solid dissolution of organic and mineral phases [117,118,119]. In other words, the interfacial phenomena are considered to be the formation process of the HA surface layer that affects the hydration layer and cell adhesion. In particular, the inclusion of SiO_4_^4−^ and CO_3_^2−^ ions in HA induces structural defects and increases its dissolution rate in vivo [120,121,122]. This promotes hydrogen bonding networks, increases cell and bone attachment rates, and activates osteoblasts, leading to the activation of surrounding tissues based on increased rates of ion solubilization and diffusion. This phenomenon is related to the ion exchange behavior at the non-apatitic layer of the HA surface, where the other elemental ions that have leached and diffused into the layer by the ion exchange increase the genetic markers and proteins for inducing osteogenesis, and some reports suggest the increase in the activity of osteoblasts [54,55,56,57,58]. These surface phenomena of ion-substituted HA affect the hydration layer, leading to significant changes in the dissolution properties of HA and contributing to its biocompatibility, resulting in improved protein adsorption, cell adhesion, and osteogenesis. 

## 4. Conclusions

In this review, the current status of the effect of substitution ions on the surface properties and biocompatibility of HA was discussed. Changing the chemical composition and crystalline structure of HA depending on the synthesis method and added ions was emphasized, which improved the surface properties of HA and thus affected its biocompatibility. In particular, the ability of HA to be substituted by silicate and carbonate is remarkable for its use as a bioceramic. Furthermore, the characteristics of the HA surface layers (i.e., hydrated and non-apatite layers) that affect subsequent biological responses were clearly summarized and categorized. Since the surface layers are important for both protein adsorption and cell adhesion, analysis of their properties may provide important clues to gain insight into the efficient mechanisms of bone formation.

## Figures and Tables

**Table 1:** Classification of the calcium phosphate compounds.

CP Compound	Abbreviation	Chemical Formula	Ca/P
Dicalcium phosphate dehydrate	DCPD	Ca (HPO_4_)_2_∙H_2_O	1.00
Octacalcium phosphate	OCP	Ca_8_H_2_(PO_4_)_6_∙5H_2_O	1.33
Tricalcium phosphate	α-TCP β-TCP	Ca_3_(PO_4_)	1.50
Hydroxyapatite	HA	Ca_10_(PO_4_)_6_(OH)_2_	1.67
Amorphous calcium phosphate	ACP	CaHPO_4_∙nH_2_O	–

**Table 2:** Different ionic elements substituted into the HA structure for improving its functions.

Substitution Ion		Function
Cation	Na^+^	Excellent osteoconductivity, improvement of cell proliferation
	K^+^	Improvement of thermal stability
	Mg^2+^	Enhancement of crystallization, crystal growth, thermal stability, influence on the dissolution
	Sr^2+^	Inhibition of bone resorption, enhancement of bone formation
	Mn^2+^	Cell adhesion activation
	Zn^2+^	Enhancement of bone formation
Anion	SiO_4_^4−^	Enhancement of bioactivity, improvement of dissolution speed
	CO_3_^2−^	Higher specific surface area, lower crystallite size, excellent osteoconductive properties, higher solubility
	F^−^	Higher stability, lower solubility, promotion of remineralization
	Cl^−^	Excellent osteoconductive properties, higher solubility

## Data Availability

Not applicable.
